# Ferroptosis: Opportunities and Challenges in Treating Endometrial Cancer

**DOI:** 10.3389/fmolb.2022.929832

**Published:** 2022-07-01

**Authors:** Jianfa Wu, Li Zhang, Suqin Wu, Zhou Liu

**Affiliations:** ^1^ Department of Gynecology, Shanghai University of Medicine and Health Sciences Affiliated Zhoupu Hospital, Shanghai, China; ^2^ Department of Gynecology, Shanghai University of Medicine and Health Sciences, Shanghai, China

**Keywords:** ferroptosis, prognosis, progress, treatment, initiation, endometrial cancer

## Abstract

Ferroptosis, a new way of cell death, is involved in many cancers. A growing number of studies have focused on the unique role of ferroptosis on endometrial cancer. In this study, we made a comprehensive review of the relevant articles published to get deep insights in the association of ferroptosis with endometrial cancer and to present a summary of the roles of different ferroptosis-associated genes. Accordingly, we made an evaluation of the relationships between the ferroptosis-associated genes and TNM stage, tumor grade, histological type, primary therapy outcome, invasion and recurrence of tumor, and accessing the different prognosis molecular typing based on ferroptosis-associated genes. In addition, we presented an introduction of the common drugs, which targeted ferroptosis in endometrial cancer. In so doing, we clarified the opportunities and challenges of ferroptosis activator application in treating endometrial cancer, with a view to provide a novel approach to the disease.

## Introduction

Endometrial carcinoma is one of the most common gynecological malignancies. In the United States, there were 66,570 new cases in 2021, of which, 12,940 patients died from endometrial cancer (Siegel et al., 2022). Most endometrial cancers are diagnosed at an early stage, and in most cases, the 5-year survival rate was over 80%; however, we must admit that the prognosis of those who have recurrence or distant metastasis was still not optimistic, and that the 5-year survival rate is only 17.8% (Jeppesen et al., 2016; European Comission 2020). Many signaling pathways have been considered to be involved in the development of endometrial cancer, such as mitogen activated kinase-like pathway (MAPK), DNA repair process, PI3K-Akt pathway, steroid hormone receptors-associated pathway, WNT pathways, L1 cell adhesion molecule interaction pathway (L1CAM), and ferroptosis pathway (López-Janeiro et al., 2021). Of them, targeting ferroptosis signaling pathway has been considered as a new therapeutic strategy for the treatment of endometrial cancer. However, it is still unclear how these signaling pathways, especially ferroptosis pathway, modulate the initiation, metastasis, treatment, and prognosis of endometrial cancer.

Ferroptosis, a new and iron-dependent cell death form, is different from apoptosis, autophagy, and necrosis. Mainly, ferroptosis plays an important role in amino acid metabolism, oxidative stress, and iron metabolism, which is involved in various physiological and pathological processes, such as neuronal degeneration, antiviral immune response, ischemia re-perfusion injury, and especially in tumor suppression (Chen et al., 2021a; Hoy et al., 2021; Xiong et al., 2021; Lei et al., 2022). Studies have found that ferroptosis is closely associated with liver cancer, stomach cancer, pancreatic cancer, breast cancer, stomach cancer, and ovarian cancer (Wang et al., 2021a; Jiang et al., 2021; Lin et al., 2021; López-Janeiro et al., 2021; Yang L et al., 2021; Yuan Y. et al., 2021). Ferroptosis activation has been considered to be a new approach to most tumors (Eling et al., 2015; Sun et al., 2016; Roh et al., 2017; Zhou et al., 2019; Gao et al., 2020). In particular, a growing number of studies have focused on the relationship between ferroptosis and endometrial cancer in recent years. Up to now, a large number of studies on ferroptosis *in vivo* and *in vitro* have provided new insights into the initiation, metastasis, recurrence, treatment, and prognosis of endometrial cancer.

In the current review, we systematically explored the relationship between ferroptosis and the initiation, metastasis, recurrence, treatment, and prognosis of endometrial cancer, in order to provide evidence-based guidance for the diagnosis and treatment of endometrial cancer.

## Ferroptosis and Initiation of Endometrial Cancer

**FIGURE 1:**
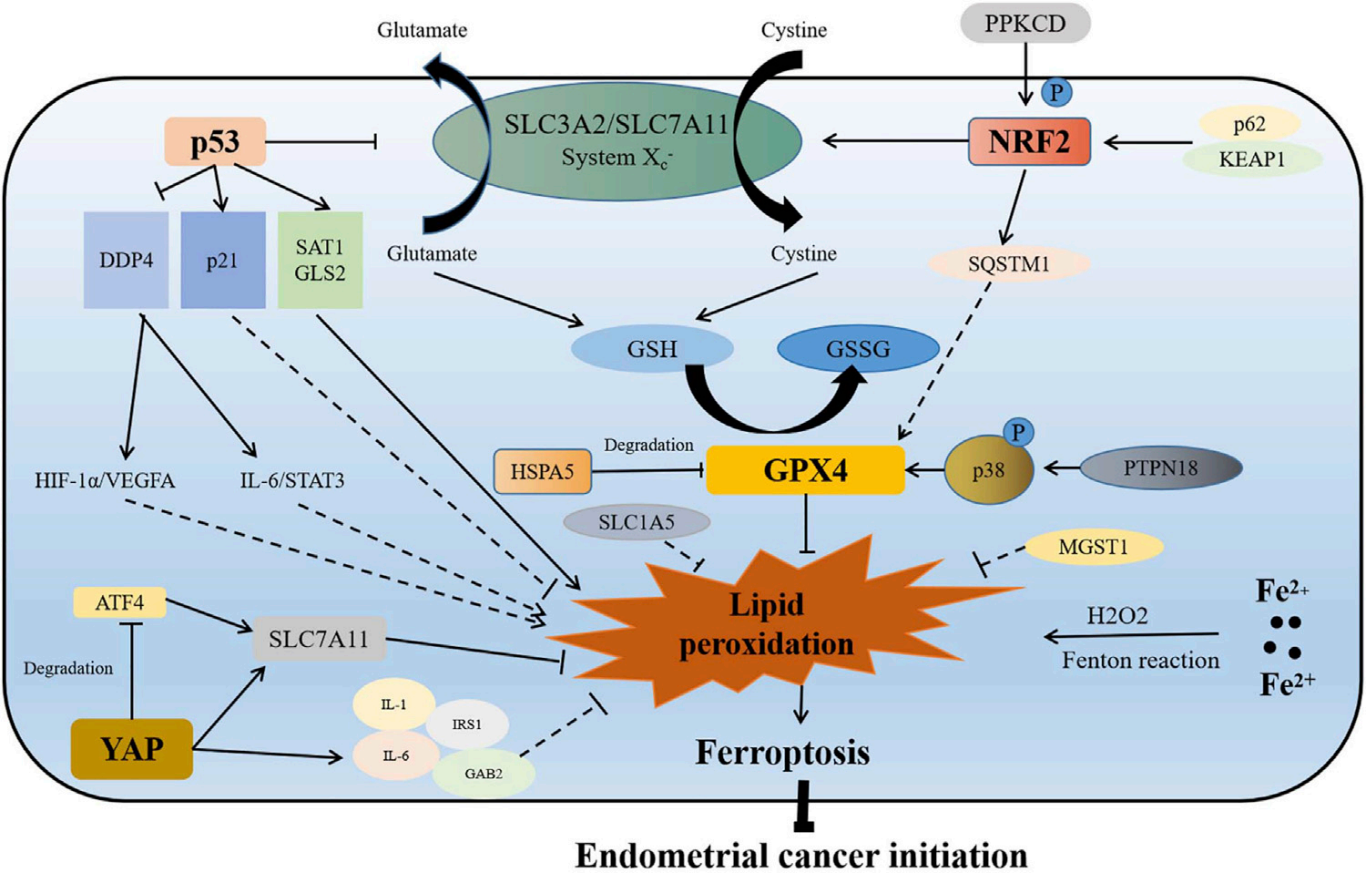
Association of ferroptosis with initiation of endometrial cancer. On the one hand, p53 directly reduces the production of GSH by inhibiting the function of the SLC3A2/SLC7A11 complex, which inhibits the function of GPX4 to promote lipid peroxidation and ferroptosis. On the other hand, p53 indirectly targets p21, SAT1, GLS2, and DDP4, which may regulate ferroptosis and endometrial cancer initiation by activating the HIF-1α/VEGFA and IL-6/STAT3 signaling pathways. When Nrf2 is overexpressed or phosphorylated activated by p62/KEAP1 or PPKCD, it inhibits lipid peroxidation and ferroptosis by promoting the expression of SQSTM1, and enhancing the function of SLC7A11 and GPX4. GPX4 is a ferroptosis inhibitor, which is regulated by HSPA5 and PTPN18. GPX4 plays an important role in inhibiting ferroptosis and promoting initiation of endometrial cancer. Increase of intracellular iron results in lipid peroxidation and ferroptosis by Fenton reaction. MGST1, SLC1A5, and YAP are also inhibitors of ferroptosis. YAP promotes the expression of SLC7A11 directly or through inhibiting ATF4 degradation. YAP also promotes the increase of IL-6, IL-1, IRS1, and GAB2, which may inhibit lipid peroxidation to promote endometrial cancer initiation. P in the graph represents phosphorylation.

Iron, an important component of most metabolic enzymes, is involved in mitochondrial oxidative phosphorylation, DNA synthesis, and cell cycle (Chen et al., 2017). Abnormal accumulation of intracellular iron is an important reason for ferroptosis, which has been considered to be associated with many gynecological diseases, such as endometrial hyperplasia, endometriosis, and repeated transplantation failure (Bielfeld et al., 2019; Ng et al., 2020; Vogt et al., 2021). Similarly, ferroptosis has been found to be involved in the initiation of endometrial cancer through different pathways (Figure 1).

Nrf2 is a prerequisite for spheroid formation *via* regulation of ferroptosis in 3D culture models (Takahashi et al., 2020). Activation of antioxidant stress signaling pathway regulated by Nrf2 is an important reason for ferroptosis resistance (Chen et al., 2021a). Nrf2 inhibits ferroptosis mainly by activating iron metabolism-related genes (SLC40A1 and MT1G), GSH metabolism-related genes (SLC7A11 and GCLM), and ROS detoxification enzymes (AKR1C1 and NQO1) in many cancers (Chen et al., 2021b). As previously revealed, the p62-Keap1-Nrf2 signaling pathway played an important role in promoting estrogen-induced endometrial hyperplasia by inhibiting ferroptosis (Zhang M et al., 2021). On one hand, Nrf2 regulated the expression of glutathione-dependent lipid antioxidant (GPX4) directly or indirectly, while GPX4 overexpression resulted in ferroptosis inhibition (Fan et al., 2017); on the other hand, the overexpression of Nrf2 was found to promote the expression of solute carrier family 7 member 11 (SLC7A11) and increase the GSH level to inhibit ferroptosis (Song and Long, 2020). The phosphorylation activation of Nrf2 with protein kinase C delta (PRKCD) contributed to endometrial hyperplasia *via* promoting sequestosome 1 (SQSTM1) expression (Feng et al., 2017). All these results suggest that Nrf2, as an important inhibitor of ferroptosis, plays an important role in the initiation of endometrial cancer. Furthermore, the positive expression rate of Nrf2 was found to be higher in endometrial serous carcinoma than in endometrioid carcinomas and clear cell carcinomas (68 vs. 6 vs. 13%) (Chen et al., 2010). In addition, Nrf2 could be used as a diagnostic marker for the different types of endometrial cancer.

Glutathione-dependent lipid antioxidant (GPX4), a member of the glutathione peroxidase family, catalyzed the reduction of hydrogen peroxide to protect cells against oxidative damage (Xu et al., 2021). GPX4, a key suppressor of the ferroptosis pathway, has been reported to be associated with many tumors (Lu Y. et al., 2021; Sha et al., 2021; Sun et al., 2021). As to endometrial cancer, proteomic analysis indicated that the expressions of GPX4, glutathione synthetase (GSS), ferroptosis suppressor protein 1 (FSP1), and transferring receptor 1 protein (TFRC) were higher in the early-stage endometrial cancer than in the normal tissues (López-Janeiro et al., 2021). Therefore, GPX4-suppressed ferroptosis can be an important reason for the initiation of endometrial cancer. Moreover, GPX4 has been recognized as a potential target for many genes; heatshock 70-kDa protein 5 (HSPA5) bound to GPX4 and inhibited its protein degradation, thus promoting the initiation of endometrial cancer (Zhu et al., 2017), and protein tyrosine phosphatase non-receptor type 18 (PTPN18) targeted and activated the p-P38-GPX4/xCT signaling pathway, which also contributed to the initiation of endometrial cancer (Wang et al., 2021a).

p53 is a double-edged sword for ferroptosis, which regulates ferroptosis through both canonical and non-canonical ferroptosis pathways (Liu and Gu 2022). However, p53 alone does not induce ferroptosis directly. p53 is an important regulator for lipid, amino acid, glucose, nucleotide, and iron metabolism (Liu and Gu et al., 2021; Liu J. et al., 2021). Based on metabolism targets, p53 contributed to ferroptosis (Liu and Gu 2022). In most cases, p53 acts as a promoter of ferroptosis. However, in some special cases, p53 can inhibit apoptosis. p53 has been identified as a central regulator of ferroptosis, which represents an independent pathway as GPX4-based ferroptosis. As an activator of ferroptosis in endometrial cancer (León-Castillo et al., 2020; Liu et al., 2020), p53 hampers SLC7A11 expression to induce ferroptosis. As an important component of cystine transport protein Xc^−^ (system Xc^−^), SLC7A11 has been found to inhibit ferroptosis by promoting cystine transport, increasing the intracellular cysteine level and GSH level (Jiang et al., 2015; Koppula et al., 2021), and also SLC7A11 has been considered to be a poor prognosis factor for endometrial cancer (Martin et al., 2022). Moreover, it was another way for p53 to induce ferroptosis when spermidine/spermine N1-acetyltransferase 1 (SAT1) and glutaminase 2 (GLS2) expressions was promoted (Kang et al., 2019). As a regulator of polyamine metabolism, SAT1 acted as a target of p53, being responsible for oxidative stress (Thomas and Thomas 2003), while SAT1 deletion weakened ferroptosis induced by p53 (Ou et al., 2016). GLS2, a member of mitochondrial glutaminases, also served as a target of p53 (Hu et al., 2010). When GLS2 was knocked down, ferroptosis caused by p53 was also minimized (Gao et al., 2015). Another important role of p53 in endometrial cancer was when cyclin-dependent kinase inhibitor 1 A (CDKN1A/p21) expression was promoted by p53. CDKN1A mutation induced microsatellite instability (MSI) *via* the epigenetic silencing of the mutL homolog 1 (MLH1) (Waheed et al., 2019). However, the relationship between p21 and endometrial cancer remains controversial. Some reports indicated that the growth of endometrial cancer cells was hampered when CDKN1A was upregulated, which implied that p21 inhibited the proliferation of endometrial cancer (Waheed et al., 2019; Costa et al., 2021). Nevertheless, Planagumà et al. (2006) found that the expression of p21 in endometrial cancer was higher compared to normal control, simple hyperplasia endometria, and complex hyperplasia endometria, which suggested that p21 promoted the initiation of endometrial cancer. Therefore, the relationship between p21 and initiation of endometrial cancer still needs to be verified by subsequent experiments. As an inhibitor of ferroptosis in endometrial cancer, another mechanism of ferroptosis regulation mediated by p53 was when dipeptidyl peptidase 4 (DPP4) expression was inhibited (Kang et al., 2019). DPP4, an intrinsic type II transmembrane glycoprotein, was found to be involved in insulin metabolism, immune regulation, and cancer development (Xie et al., 2017). In endometrial cancer, DDP4 is a risk factor, which promotes cancer proliferation, invasion and tumorigenesis through HIF-1a/VEGFA signaling, and IL-6/STAT3 signaling pathway. However, DDP4 inhibitor therapy has been reported to be capable of inhibiting tumor growth (Yang et al., 2017; Yang et al., 2021a).

As to iron, which is an important condition for ferroptosis, its dietary intake was positively associated with endometrial cancer risk (adjusted OR = 1.9; 95% CI = 1.4–2.7), especially in postmenopausal women (OR = 2.2; 95% CI = 1.4–3.4) and women with BMI ≥25 kg/m^2^ (OR = 3.2; 95% CI = 1.4–7.5) (Kallianpur et al., 2010). A previous study, which enrolled 60,895 women in the Swedish mammography cohort, indicated that the higher intake of heme iron mildly increased the risk of endometrial cancer (RR: 1.24; 95% CI: 1.01–1.53; for ≥1.63 mg/d vs. <0.69 mg/d), so did the higher intake of total iron (RR: 1.31; 95% CI: 1.07–1.61; for ≥15.09 mg/d vs. <12.27 mg/d) (Genkinger et al., 2012). However, controversy still exists between dietary iron and endometrial cancer, as indicated by a large cohort study in Canada with 34,148 women enrolled and followed for a mean of 16.4 years, showing that there was no association between intake of meat or any of the dietary iron-related variables and risk of endometrial cancer. Furthermore, iron overload caused by increased iron absorption reduced iron storage and restricted iron outflow contributed to ferroptosis (Kabat et al., 2008). On one hand, increased intracellular iron promoted the increase of reactive oxygen species (ROS) through iron-dependent Fenton reaction (Chen et al., 2020). On the other hand, iron-containing lipid oxidase was activated to induce lipid peroxidation (Stockwell 2017). Nevertheless, iron-chelating agents (deferoxamine), as well as drugs which increased iron-mediated toxicity (sulfasalazine, statins, sorafenib, *etc.*) showed favorable effects in many cancers (Stockwell 2017). In particular, the combination of sulfasalazine and cisplatin indicated synergistic inhibitory effect on cell proliferation in uterine serous carcinoma cell lines (Sendo et al., 2022).

Other ferroptosis-associated genes have also been found to be involved in the initiation of endometrial cancer (Supplementary Table S1). Since microsomal glutathione S-transferase 1 (MGST1) is a ferroptosis suppressor, the expression of MGST1 was higher in endometrial cancer than in the normal tissues (Yan et al., 2022). The upregulation of solute carrier family 1 member 5 (SLC1A5), a glutamine transporter, has also been observed in many cancers (Huang et al., 2014; Kaira et al., 2015; Luo et al., 2018). In endometrial cancer in comparison with the normal endometrium, highly expressed SLC1A5 was similarly found in endometrioid and serous subtypes of endometrial carcinoma (Marshall et al., 2017). As a novel ferroptosis inducer, BRCA1-associated protein 1 (BAP1) encodes a nuclear deubiquitinating enzyme. BAP1 represses SLC7A11 expression by decreasing H2Aub occupancy on the SLC7A11 promoter in many cancers (Zhang et al., 2018). Nevertheless, BAP1 was found to be rarely investigated in endometrial cancer. One case report revealed that the negative expression of BRCA1-associated protein 1 (BAP1) was observed in the peritoneal masses after endometrial cancer surgery (Makiuchi et al., 2020). As a ferroptosis suppressor, Yes1 associated transcriptional regulator (YAP) is also a downstream gene of the Hippo signaling pathway. On one hand, YAP/TAZ directly induced the expression of SLC7A11; on the other hand, it sustained the protein stability of ATF4, which synergistically induced SLC7A11 expression to inhibit ferroptosis (Gao et al., 2021). Moreover, Wu et al. (2019) reported that the NF2-YAP signaling pathway played an important role on ferroptosis suppression, while antagonizing this signaling pathway contributed to ferroptosis through upregulating expression of Acyl-CoA Synthetase long-chain family member 4 (ACSL4) and TFRC The expression of YAP was higher in endometrial cancer than in the normal tissues and cells, which was associated with higher grade, stage, lympho-vascular space invasion, and postoperative recurrence/metastasis (Tsujiura et al., 2014; Cheng et al., 2020); the inhibition of YAP restrained proliferation, increasing therapy sensibility by reducing interleukin-6 (IL-6), IL-11, and IRS1 (Wang C. et al., 2016; Wang et al., 2019); and the knockdown of YAP and TAZ also prevented PI3K pathway activation by inhibiting the expression of GAB2 linker molecule in endometrial cancer (Wang et al., 2017).

However, most of the previous studies have been based on *in vitro* experiments or correlational studies, with a lack of large-sample clinical studies; most of the mechanism clarifications have not been sufficient enough. The underlying mechanism of ferroptosis-associated genes still needs to be further explored to better understand the initiation of endometrial cancer.

## Ferroptosis and Metastasis or Recurrence of Endometrial Cancer

Endometrial cancers metastasize mainly through lymphatic metastasis and local invasive metastasis, but less through hematogenous metastasis. It has been reported that 71% of stage 3 patients experience distant metastasis (Tangjitgamol et al., 2004; Franchello et al., 2015), and that 15% of patients with FIGO I and II endometrial cancer undergo recurrences, especially those who had deep myometrial invasion and lympho-vascular invasion (Fung-Kee-Fung et al., 2006; Sartori et al., 2010). Since metastasis and recurrence are closely associated with prognosis of endometrial cancer, the 5-year overall survival (OS) rates of patients with metastasis, pelvic recurrence, and extrapelvic recurrence were lower than those of the localized endometrial carcinoma (16, 55 and 17% vs. 95%) (Xu et al., 2016; National Cancer Institute Surveillance, 2017). Therefore, it is of great significance to clarify the mechanism of metastasis and recurrence of endometrial cancer, since it has not been unclear so far. Thus no effective strategy is available to improve the prognosis of endometrial cancer, especially with metastasis or recurrence.

**FIGURE 2:**
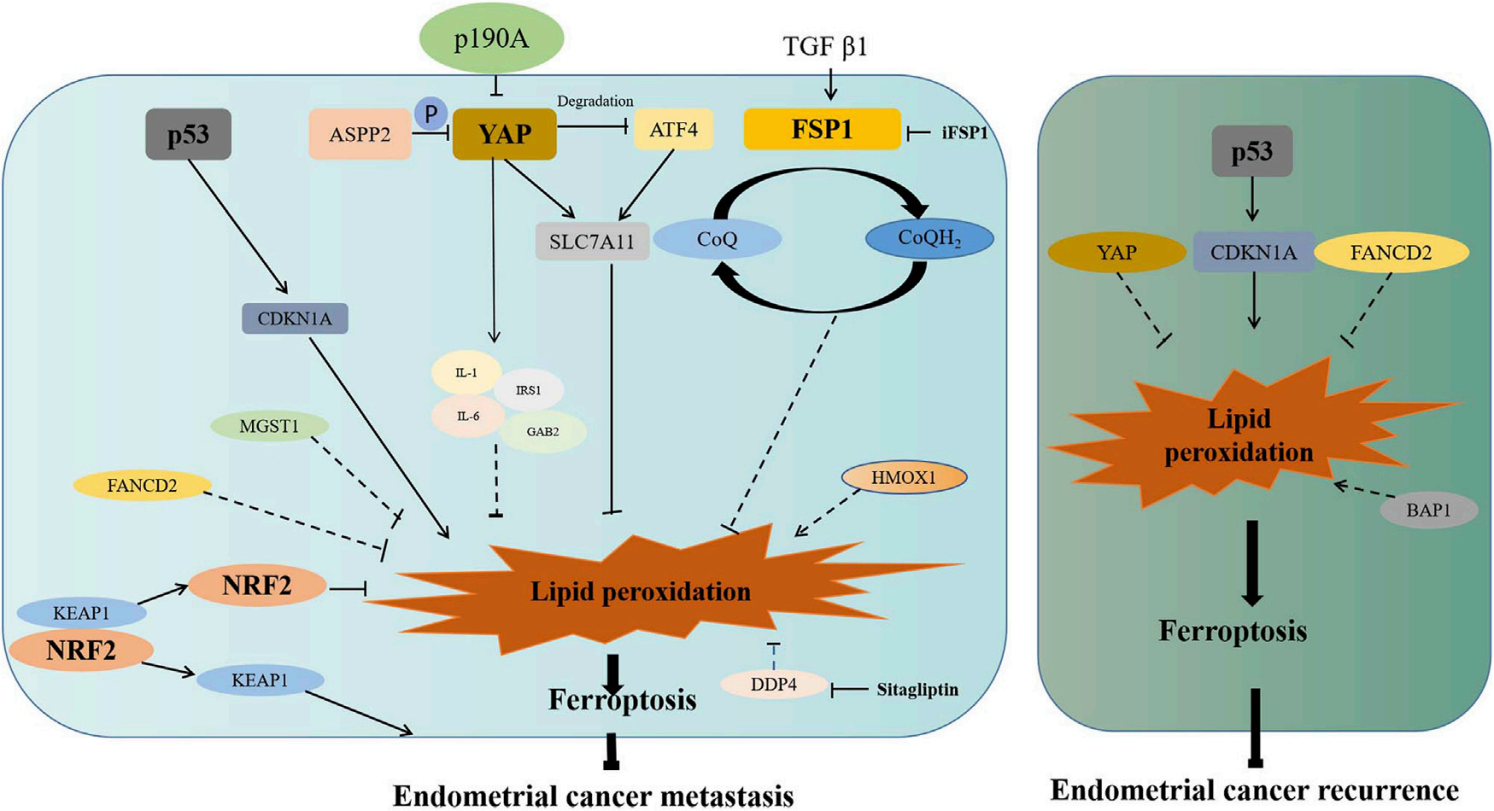
Association of ferroptosis-associated genes with metastasis or recurrence of endometrial cancer. ASSP2 and p190A inhibit migration of endometrial cancer *via* inactivating the Hippo–YAP signaling pathway. YAP promotes the expression of SLC7A11 directly or through inhibiting ATF4 degradation. YAP also promotes the increase of IL-6, IL-1, IRS1, and GAB2, which may inhibit lipid peroxidation to promote invasion or migration. TGFβ1 promotes the expression of FSP1, which inhibit lipid peroxidation to promote invasion or migration. After Nrf2 dissociated from KEAP1, activated Nrf2 inhibits lipid peroxidation to promote invasion or migration. p53 enhances the expression of CDKN1A to promote lipid peroxidation. MGST1, FANCD2, and DDP4 may promote invasion or migration through inhibiting lipid peroxidation and ferroptosis. HOMX1 may restrain invasion or migration through promoting lipid peroxidation and ferroptosis. Moreover, increased ubiquitinated degradation of p53 results in the decreased expression of CDKN1A, which results in reduced ferroptosis and enhanced recurrence. FANCD2 and YAP may promote recurrence through inhibiting lipid peroxidation and ferroptosis. BAP1 may prevent recurrence through promoting lipid peroxidation and ferroptosis. P in the graph represents phosphorylation.

Previous studies have found that ferroptosis is associated with metastasis and recurrence of many tumors (Ubellacker et al., 2020; Li et al., 2021; Liu W. et al., 2021; Luis et al., 2021). In endometrial cancer, actually, many ferroptosis-associated genes have been discovered to be involved in its metastasis or recurrence (Figure 2). Bioinformatics analysis showed that CDKN1A was closely related to the occurrence of type II endometrial carcinoma, which was prone to recurrence and metastasis (Zhang K. et al., 2019). More importantly, DETA/NO and progesterone-inhibited invasion of endometrial cancer by upregulating CDKN1A expression *in vitro* (Dai et al., 2002; Waheed et al., 2019). As a ferroptosis suppressor, fanconi anemia complementation group D2 (FANCD2) was involved in DNA damage repair (Song et al., 2016). According to a tissue microarray analysis, FANCD2 overexpression was associated with lympho-vascular invasion in type I endometrial cancer and recurrence in type II endometrial cancer (Mhawech-Fauceglia et al., 2014). High expression of MGST1 was also found to be associated with the high frequency of tumor invasion (Yan et al., 2022). Therefore, MGST1 can serve as a predictive factor for the prognosis of endometrial cancer.

It was revealed that DDP4 facilitated the invasion of endometrial cancer *in vitro*, while this facilitation was abrogated with the DDP4 inhibitor (Yang et al., 2017). Moreover, p53 inactivated with enhanced ubiquitination was found to be associated with the invasion or recurrence of endometrial cancer (Liu et al., 2020). As an apoptosis inducer, apoptosis-stimulating p53 protein 2 (ASPP2) suppressed cell migration and invasion by reducing the expression of phosphorylated YAP (Konno et al., 2020). Since p190A is frequently mutated in endometrial cancer, its knockout was reported to promote cell proliferation and migration *via* activation of the Hippo–YAP pathway (Wen et al., 2020). Being a molecular marker of fibrosis, fibroblast-specific protein 1 (FSP1) acts as a GPX4-independent ferroptosis inhibitor. FSP1 has been reported to inhibit ferroptosis by reducing CoQ10 to prevent lipid oxidation, while cell sensitivity to ferroptosis increased after FSP1 inhibitor (iFSP1) treatment (Bersuker et al., 2019; Doll et al., 2019). In endometrial cancer, it was revealed that TGF-beta1 stimulated cell migration and invasion by increasing FSP1 expression (Xie et al., 2009).

In addition, the drugs which target ferroptosis have shown to be capable of invasion inhibition in endometrial cancer, as in the case of juglone, which promoted HMOX1 expression, thereby inhibiting the migration of endometrial cancer (Yuan Y. et al., 2021) and of simvastatin which inhibited metastasis through the modulation of the MAPK and AKT/mTOR pathways (Schointuch et al., 2014).

At present, however, the studies are still limited on the relationship between ferroptosis-associated genes and metastasis or recurrence of endometrial cancer. The previous studies are mostly based on clinical correlation analysis and *in vitro* experiments, and the mechanism of ferroptosis-associated genes has not been sufficiently clarified on the metastasis or recurrence of endometrial cancer. This, therefore, pushes us to stay at the forefront of the studies to pursue the underlying mechanism of endometrial cancer metastasis or recurrence.

## Ferroptosis and Treatment of Endometrial Cancer

In general, surgery is the main approach to endometrial cancer. To prevent its metastasis and recurrence, it is important that post-surgical adjuvant chemotherapy and radiotherapy are administered (Lu et al., 2020; Concin et al., 2021); however, it is admitted that drug resistance is a significant challenge for the treatment. Drug resistance is a complex process in endometrial cancer, which involves factors such as enhancing proliferation, reducing apoptosis, and abnormal transmembrane transport of drugs (Huang W. et al., 2021; Kong et al., 2021; Yuan S. et al., 2021). However, no good predictor is still available for drug resistance in endometrial cancer. As a newly discovered way of cell death, ferroptosis has been considered to be closely related to drug resistance in endometrial cancer (He et al., 2021). It was reported that the IC50 of cisplatin and paclitaxel was higher in those who had a low score than in those who had a high score of ferroptosis, while the IC50 of erlotinib, rapamycin, and temsirolimus was lower in those who had a low score than in those who had a high score of ferroptosis (Wang et al., 2021b). This suggests that those who had a low score of ferroptosis are more likely to be resistant to cisplatin and paclitaxel, while those who had a high score are more likely to be resistant to erlotinib, rapamycin, and temsirolimus. Similarly, another ferroptosis-related prognosis signature showed lower IC50 of roscovitine, vinblastine, tipifarnib, lapatinib, and other twenty-two routinely administered chemotherapy drugs in the low-risk group than the high-risk group (Liu J. et al., 2021). Moreover, quite a number of ferroptosis-associated genes are responsible for chemoresistance in ovarian cancer, as indicated by the activation of the HSPA5-GPX4 pathway, which induced ferroptosis resistance, an important reason for gemcitabine resistance (Zhu et al., 2017); by the overexpression of FANCD2, which resulted in platinum resistance, while restraining FANCD2 expression with pristimerin sensitized endometrial cancer to platinum (Bi et al., 2019), and the activation of the Nrf2 signaling pathway led to chemoresistance in endometrial cancer, while targeting Nrf2 with metformin rendered endometrial cancer more sensitive to chemotherapeutics (Wang Y. et al., 2016; Bai et al., 2018). In view of these evidence, the ferroptosis-associated genes serve as favorable predictors for chemotherapy sensitivity in clinical practice.

As indicated in Supplementary Table S1, targeting ferroptosis-associated genes could be an effective way for treatment in endometrial cancer. Being a natural compound, quinones are of good anti-inflammatory, antioxidant stress, and antitumor effects. Juglone and plumbagin, as natural quinones compounds, have been found to have a good therapeutic effect on endometrial carcinoma (Zhang Y. Y. et al., 2019; Zhang et al., 2020). In particular, juglone, one of the 16 organic compounds of *C. cathayensis*, induced ferroptosis by promoting intracellular iron accumulation, GSH, and MDA depletion in endometrial carcinoma (Zhang Y. Y.et al., 2021). However, the therapeutic effects of juglone and plumbagin on endometrial cancer are still limited to *in vitro* experiments. Thus, *in vivo* experiments and clinical studies are still needed to determine the therapeutic effects of juglone and plumbagin.

Known as the inducer of ferroptosis, statins target cholesterol synthesis of rate-limiting enzymes (HMG-CoA) (Schweitzer et al., 2020). FSP1-CoQ10-NAD (P) H signaling pathway acted synergistically with GPX4 and GSH to inhibit lipid peroxidation and ferroptosis (Doll et al., 2019). As a common stain, simvastatin was found to inhibit cell proliferation and induce cell death in a dose-dependent manner in endometrial cancer cell lines (Schointuch et al., 2014). Statins exerted a chemo-protective effect in endometrial cancer (Kato et al., 2010). However, there is still a lack of sufficient evidence to verify the association of statins use with prognosis improvement in endometrial cancer (Hafizz et al., 2020). Moreover, quite a number of problems and challenges are lying ahead to be addressed; this is particularly true when statins’ compatible variety, optimal dose, application duration, and therapeutic effects or side effects are to be certified while they are being combined with routinely administered chemotherapy drugs.

Sorafenib, a novel oral targeted therapy, can inhibit the serine/threonine kinase activation of RAF-1 and B-Raf, as well as the tyrosine kinase activation of vGFR-2, VEGF-3, PDGF-β, KIT, and FLT-3 receptors (Keating, 2017). Specially, it also functions as an inducer of ferroptosis to inhibit the activation of system Xc^−^(Gao et al., 2021). In the case of endometrial cancer, sorafenib was revealed to sensitize endometrial carcinoma cells to TRAIL- and Fas-induced apoptosis *in vitro* (Llobet et al., 2010). Moreover, sorafenib alone induced apoptosis in endometrial cancer by transcriptionally inhibiting myeloid cell leukemia 1 (McL-1) expression and promoting its protein degradation (Sun et al., 2013), while it showed a limited effect on both uterine carcinoma and uterine carcinosarcoma in a multi-center phase II clinical study (Nimeiri et al., 2010). Sorafenib activated MAPK/JNK-dependent autophagy to enhance the antitumor activity (Eritja et al., 2017). Nevertheless, there is still a long way to go before sorafenib can be used as a routine clinical therapy for endometrial cancer, since quite a number of problems and challenges lie ahead to be addressed in terms of clinical efficacy, adverse reaction, drug resistance and regulatory mechanisms associated with ferroptosis.

Given that the expressions of TMB and MSI in endometrial cancer are significantly correlated to the SLC7A11 level, it has been hypothesized that the use of ferroptosis inducers can have synergistic effects with immune checkpoint inhibitors (McConechy et al., 2015; Yang, 2015; Yang et al., 2019). However, this hypothesis only stays at the theoretical level, which still needs to be verified *via in vivo* and *in vitro* experiments.

In conclusion, ferroptosis inducers have shown a potential application value in the treatment of endometrial cancer, by increasing the sensitivity of cancer to the traditional medications compared to those traditional drugs such as carboplatin, paclitaxel, doxorubicin, bevacizumab, medroxyprogesterone acetate, and GnRHa. However, the application data of ferroptosis inducers is limited in clinical, especially the specific ferroptosis inducers. The efficacy and safety of ferroptosis inducers still need to be verified with further basic and clinical studies, so do the indications and applicable populations of ferroptosis inducers as well as the efficacies, drug dosages and side effects of single drug or different combination regiments. In particular, it remains unknown whether ferroptosis inducers can simultaneously induce ferroptosis of cancer cells and immune cells to comprise the body’s immune function, and how ferroptosis inducers can modulate different immune cells to enhance or weaken the body’s immune response.

## Ferroptosis and Prognosis of Endometrial Cancer

**FIGURE 3:**
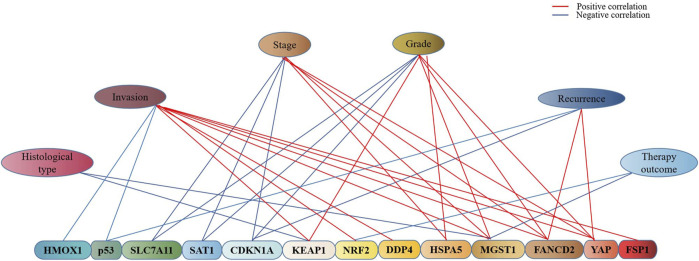
Association of ferroptosis-associated genes with clinical characteristics of endometrial cancer. As indicated in the graph, there exists an association of ferroptosis-associated genes with the stage, grade, invasion, recurrence, histopathological type, and therapy outcome of endometrial cancer, blue represents the negative and red represents the positive.

It is well recognized that the prognosis of endometrial cancer is related to quite a number of contributing factors such as tumor histological type, tumor stage, pathological stage, metabolism, and recurrence, which is also true of many ferroptosis-associated genes in endometrial cancer (Figure 3). The high expressions of CDKN1A, SLC7A11, and SAT1 were found to be linked to the low stage, grade of pTNM, and longer survival time in endometrial cancer (Qin et al., 2021). As a ferroptosis suppressor, it was revealed that FANCD2 overexpression was associated with high tumor grade, advanced tumor stage, and lympho-vascular invasion in type I endometrial cancer (Mhawech-Fauceglia et al., 2014), while in type II endometrial cancer, the patients with the positive expression of FANCD2 were found to be more likely to recur within 5 years and with poor 5-year recurrence free survival (RFS) (71.4 vs. 85.5%) and OS (68 vs. 80.3%) (Mhawech-Fauceglia et al., 2014). The expression of HSPA5 was reported to be higher in high-risk endometrial cancer than in low-risk endometrial cancer and normal endometrium, which suggested that HSPA5 was also associated with higher malignant degree and poor prognosis of endometrial cancer (Teng et al., 2013). In addition, univariate and multivariate regression analyses indicated that the high expression of MGST1 was associated with the high clinical stage (TNM), poor primary therapy outcome, poor histological type, high tumor invasion, and poor histologic grade (Yan et al., 2022), hence MGST1 is regarded as a progress predictive factor for endometrial cancer.

**FIGURE 4:**
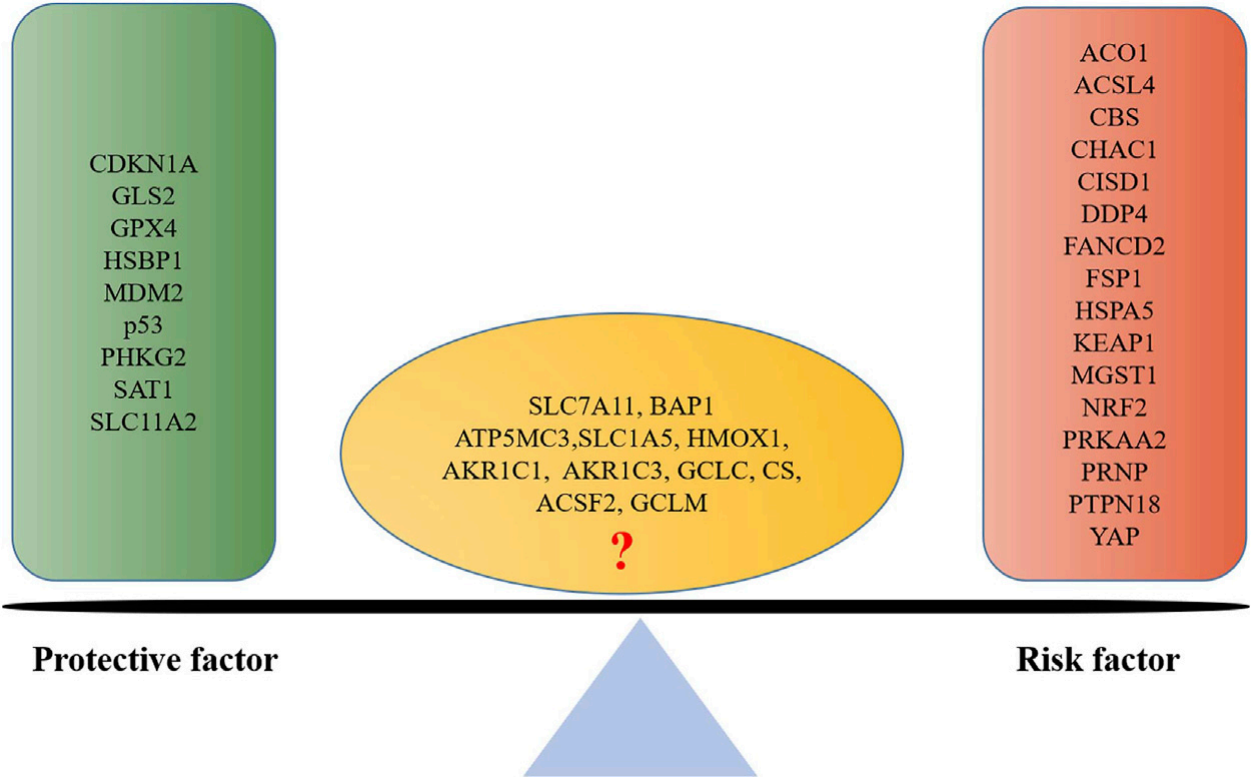
Ferroptosis-associated gene-based protective factor and risk factor in endometrial cancer. A list is made of ferroptosis-associated genes involved in endometrial cancer prognosis based on the previous literature; those which are positively associated with a good prognosis are defined as protective factors, and those which are negatively associated are defined as risk factors.

Moreover, many other ferroptosis-associated genes were also found to be associated with prognosis of endometrial cancer *via* clinical observation or bioinformatics analysis (Figure 4). A pan-cancer analysis indicated that SLC7A11, a key gene of ferroptosis, was a risk factor for worsen OS in such cancers as adrenocortical carcinoma, bladder urothelial carcinoma, head-and-neck squamous cell carcinoma, kidney renal clear cell carcinoma, liver hepatocellular carcinoma, and skin cutaneous melanoma, while it was a protective factor for prolonged OS for ovarian cancer and rectum adenocarcinoma (He et al., 2021). No association has been reported of SLC7A11 with endometrial cancer; however, the association of SLC7A11 with the prognosis of endometrial cancer remains controversial. Qin et al. (2021) reported that SLC7A11 was associated with prolonged survival time of endometrial cancer, which suggested a protective factor. While CDKN1A was found to be an activator of ferroptosis, which was significantly associated with better prognosis of endometrial cancer (Yamawaki et al., 2017); and ACSL4 was associated with lipid metabolism and lipid peroxidation dependent ferroptosis, while the low expression of ACSL4 was observed in endometrial cancer to be associated with better prognosis (Yu et al., 2022).

Intriguingly, quite a few research studies have probed into the regulated mechanism of ferroptosis-associated genes in endometrial cancer, as indicated by the evidence that ferroptosis-associated genes were positively related to M1 macrophages, M2 macrophages, T cell follicular helper, and B cells naive, while they were negatively related to NK cells activated, T cells regulatory (Tregs) and neutrophils (Liu W. et al., 2021); and that damage-associated molecular patterns (DAMPS) released by ferroptosis were sensed by the immune cells, thus enhancing inflammatory responses and improving the immune microenvironment in cancer was found (López-Janeiro et al., 2021). Therefore, it was thought that different patients with endometrial cancer could have different immune microenvironments to have different prognosis (Blaisdell et al., 2015; Antomarchi et al., 2019; Pan et al., 2019). An abnormal microenvironment induced by ferroptosis-associated genes can be the underlying mechanism of poor prognosis in endometrial cancer.

In fact, gene-combined panels have been used to predict the prognosis of endometrial cancer. Of them, TCGA molecular typing and ProMisE molecular typing, based on POLE gene, p53 gene, and DNA mismatch repair genes, are the most classic molecular typing of endometrial cancer (Levine et al., 2013; Eriksson et al., 2021). Definitely, these molecular typings established play an important role in predicting prognosis and guiding clinical practice; however, two of them still have shortcomings, which limits their clinical application: complicated testing processes and high testing cost, and their consistency with clinical practice still needs to be improved. Therefore, it is significant that a novel, simple, and economical molecular prognostic model be explored in predicting endometrial cancer. It is well known that abnormal ferroptosis is an important reason behind the poor prognosis of endometrial cancer, as indicated by the evidence that molecular typing based on ferroptosis-associated genes showed good prognosis predictive value: 1) the ferroptosis score, based on thirteen ferroptosis-associated genes, was established, and OS of patients with a low score of ferroptosis was superior to that of those with high score of ferroptosis (AUC = 0.726) (Wang et al., 2021b); 2) a molecular typing of endometrial cancer, based on six ferroptosis-associated genes of HMOX1, KEAP1, HSBP1, SAT1, CISD1, and GPX4, showed good 1-, 3-, and 5-year prognostic predictive value (AUC = 0.705, 0.676, and 0.713) (Liu J. et al., 2021); 3) a ferroptosis-associated gene signature with eight genes of MDM2, GPX4, PRKAA2, PRNP, SLC11A2, ATP5MC3, PHKG2, and ACO1, showed better 1-, 3-, and 5-year prognostic predictive value (AUC = 0.676, 0.797, and 0.826) than the aforementioned two ferroptosis prognosis molecular typings (Weijiao et al., 2021). Ferroptosis prognosis molecular typing possessed a comparable or superior prognosis predictive value when compared with the previous prognosis molecular typings of endometrial cancer (Table 1) (Tang et al., 2019; Yang et al., 2021b; Coll-de la Rubia et al., 2021; Huang S. et al., 2021; Lu N. et al., 2021; Pang et al., 2021; Wang Z. et al., 2021). As indicated by the evidence, ferroptosis-associated genes-based molecular typing can be considered as an effective method to predict the prognosis of endometrial cancer.

**TABLE 1:** Molecular typing for endometrial cancer.

Author	Article title	Date	Gene panel	Risk prediction formula	AUC
Yin Weijiao et al.	Immune infiltration and a ferroptosis-associated gene signature for predicting the prognosis of patients with endometrial cancer	2021	MDM2, GPX4, PRKAA2, PRNP, SLC11A2, ATP5MC3, PHKG2, and ACO1	Risk score = (–0.34216 × MDM2 expression) + (–0.08952 × GPX4 expression) + (0.55497 × PRKAA2 expression) + (0.08230 x PRNP expression) + (– 0.46253 × SLC11A2 expression) + (0.41109 × ATP5MC3 expression) + (–0.50883 × PHKG2 expression) + (0.30930 × ACO1 expression)	1-year: 0.676
					2-year: 0.775
					3-year: 0.797
					5-year: 0.826
Jinhui Liu et al.	Identification of the prognostic signature associated with tumor immune microenvironment of uterine corpus endometrial carcinoma based on erroptosis-related genes	2021	HMOX1, KEAP1, HSBP1, SAT1, CISD1, and GPX4	Risk score = = (0.002907 × HMOX1) + (0.013486 × KEAP1) + (−0.089640 × HSBP1) + (−0.001665 × SAT1) + (0.148,239 ×CISD1) + (−0.003060 × GPX4)	1-year: 0.705
					3-year: 0.607
					5-year: 0.713
L.S. E. ERIKSSON et al.	Combination of proactive molecular risk classifier for endometrial cancer (ProMisE) with sonographic and demographic characteristics in preoperative prediction of recurrence or progression of endometrial cancer	2021	ProMisE subtype, age, waist circumference , sonographic tumor extension, and size	-	3-year: 0.890
Xiao Yang et al.	A novel transcription factor-based prognostic signature in endometrial cancer: establishment and validation	2021	MSX1, HOXB9, E2F1, DLX4, BNC2, DLX2, PDX1, POU3F2, and FOXP3	Risks core=(-0.0621*ExpDLX2)+ (-0.2395* ExpFOXP3)+(0.1016*ExpPOU3F2)+(0.2536*ExpPDX1)+(0.3276*ExpBNC2)+(0.2091*ExpDLX4)+(0.0158*ExpE2F1)+(0.0071*ExpHOXB9)+(-0.0021*ExpMSX1)	5-year: 0.761
Xuecheng Pang et al.	Development and validation of m6A regulators’ prognostic significance for endometrial cancer	2021	IGF2BP1 and YTHDF3	Risk score = 0.0904*IGF2BP1 + 0.195* YTHDF3	1-year:0.6552
					3-year:0.6408
					5-year:0.6439
Nan Lu et al.	MiRNA-based tumor mutation burden diagnostic and prognostic prediction models for endometrial cancer	2021	hsa-miR-146a-5p, hsa-mir-708-5p, hsa-miR-4746-5p, hsa-miR-452-5p, hsa-miR-452-3p, hsa-miR-224-5p, hsa-miR-375-3p, hsa-miR-30a-5p, hsa-miR-598-3p, hsa-miR-335-3p, hsa-miR-30c-5p, hsa-miR-101-5p, hsa-miR-210-3p ,hsa-miR-676-3p, hsa-miR-130a-3p, hsa-miR-1266-5p, hsa-miR-1271-5p ,hsa-miR-130a-5p, hsa-miR-203b-3p, hsa-mir-3074-5p, and hsa-miR-30d-5p	Risk score = hsa-miR-146a-5p * 0.091672 + hsa-mir-708-5p * (−0.01454) + hsa-miR-4746-5p *0.647,021 + hsa-miR-452-5p * 0.057283 + hsa-miR-452-3p * (−0.26965) + hsa-miR-224-5p * 0.018647 + hsa-miR-375-3p * 0.11641 + hsa-miR-30a-5p * 0.328,458 + hsa-miR-598-3p * 0.022044 + hsa-miR-335-3p * (−0.49775) + hsa-miR-30c-5p * (−0.63721) + hsa-miR-101-5p * 0.021696 + hsa-miR-210-3p * 0.571,997 + hsa-miR-676-3p * (−0.53052) + hsa-miR-130a-3p * 0.021182 + hsa-miR-1266-5p * 0.346,479 + hsa-miR-1271-5p * (−0.10099) + hsa-miR-130a-5p * (−0.09051) + hsa-miR-203b-3p * (−0.06494) + hsa-mir-3074-5p * 0.432,032 + hsa-miR-30d-5p * (−0.40024)	1-year: 0.649
					3-year: 0.602
					5-year: 0.699
Ziwei Wang et al.	An immune-related long noncoding RNA signature as a prognostic biomarker for human endometrial cancer	2021	ELN-AS1, AC103563.7, PCAT19, AF131215.5, LINC01871, AC084117.1, NRAV, SCARNA9, AL049539.1, POC1B-AS1, AC108134.4, and AC019080.5	Risk Score= (ELN-AS1 × 0.229) + (AC103563.7 × 0.313) + (PCAT19× −0.277) + (AF131215.5 × 0.252)+ (LINC01871 × −0.357) + (AC084117.1 × 0.449) + (NRAV × −0.433) + (SCARNA9 × −0.339) +(AL049539.1 × 0.476) + (POC1B-AS1 × −0.758) + (AC108134.4× −0.262) + (AC019080.5 × 0.899)	3-year: 0.808
					5-year: 0.831
Shijin Huang et al.	Identification of a four-gene signature with prognostic significance in endometrial cancer using weighted gene correlation network analysis	2021	BUB1B, NDC80, TPX2, and TTK	Risk Score = 0.8871 × expression of TTK + (−0.5266 × expression of BUB1B) + (−0.5022 × expression of NDC80) + 0.5177 × expression of TPX2	2-year: 0.683
					3-Year:0.703,
					5-year: 0.684
Eva Coll-de la Rubia et al.	*In silico* approach for validating and unveiling new applications for prognostic biomarkers of endometrial cancer	2021	ASRGL1, ESR1, FASN, HDGF, MACC1, MCM6, MCM7, MSH2, MSH6, PTK2, and TPX2	—	4-year:0.827

However, most studies on the correlation of ferroptosis-associated genes with the prognosis of endometrial cancer have been conducted based on bioinformatics analysis, lacking large, and multi-center clinical samples for prospective validation. The sensitivity, specificity, and stability of ferroptosis-associated genes-based molecular typing models still merit further investigations. The correlation of ferroptosis-associated genes with immune response, immune infiltration still remains unclear, and the previously reported studies have been performed mostly based on correlation analysis of clinical cases. Whether there is a causal or concomitant correlation between the abnormal expression of ferroptosis-associated genes with the immune response and immune infiltration in endometrial cancer still needs to be verified by a large number of experiments *in vivo* and *in vitro*. More importantly, it remains unclear whether the use of ferroptosis inducer will improve the prognosis of endometrial cancer; further research studies are needed in terms of dosage selection, application method, and therapeutic safety and effectiveness.

## Future Researches

Further studies are needed on the association of ferroptosis with initiation, metastasis, recurrence, treatment, and prognosis of endometrial cancer. A focus is to be placed on the identification of the key ferroptosis-associated genes in endometrial cancer. Much importance is to be attached to the underlying mechanism of ferroptosis-associated genes in the initiation and progress of endometrial cancer, with the laboratory-derived research results shifting to the clinical investigations to determine proper ferroptosis inducers and precise dose and duration of administration. In so doing, the best treatment plan, either as combination chemotherapy or non-chemotherapy, can be developed for the patient with endometrial cancer.

## Conclusion

A large amount of evidence suggest that ferroptosis is involved in all aspects of endometrial cancer, including initiation, metastasis, recurrence, treatment, and prognosis. Ferroptosis-associated gene-based molecular typing model has shown a comparable prognosis predictive value than others, and many conventional drugs, which activate ferroptosis, have also shown favorable antitumor effects *in vitro*. Targeting ferroptosis has displayed a favorable role in reversing drug resistance of endometrial cancer. Therefore, it is hypothesized that targeting ferroptosis can be an underlying therapeutic approach to endometrial cancer, although the evidence is not sufficient enough at present.
