# Supplementary material for: Ferroptosis: Opportunities and Challenges in Treating Endometrial Cancer
Source: Front Mol Biosci. 2022 Jul 1;9:929832. doi: 10.3389/fmolb.2022.929832 (PMC9284435; doi:10.3389/fmolb.2022.929832)
Supplement: Supplementary file 1 [file Table1.docx]

**Table 1. Studies on ferroptosis in endometrial cancer**

| Author | Name of drugs | Targeted gene | Modulator | In vitro  (cell lines) | In vivo | Clinical trial | Mechanisms/Effectiveness |
| --- | --- | --- | --- | --- | --- | --- | --- |
| Jianling Bi et al.  Kanako Sendo, et al.*  Yiying Wang et al.  Mingzhu Bai et al.  Lifeng Feng, et al.*  Zhang M, et al.*  Yuan-Yuan, et al.  Yuan-Yuan, et al.*  X. S. Zhang et al.  Monica N Schointuch, et al.  Kato S et al.  D Llobet et al.  Nian-Kang Sun et al.  Halla S. Nimeiri et al.*  A D Marshall, et al.*  Sana Waheed, et al.  Xiaoqing Yang, et al.*  Haibo Wang, et al.*  Jacinta H Martin, et al.*  Kaoru Yamawaki, et al.  Xiaoqing Yang, et al.*  Yan Liu, et al.*  Jing Wang, et al.  Takumi Konno, et al.  Xiaoli Wen, et al.  Yun Cheng, et al.  Masahiro Tsujiura, et al.  Chao Wang,et al.*  Chao Wang, et al.*  Ran Xie, et al.*  Yincheng Teng, et al.  Ning Chen, et al.  Asha R Kallianpuret al.  G C Kabat, et al.*  Jeanine M Genkinger, et al.*  Feng-Hsiang Tang, et al.  [Hao Wang](https://pubmed.ncbi.nlm.nih.gov/?term=Wang+H&cauthor_id=34650597), et al.  Jiajun He, et al.  [Jianing Yan](https://pubmed.ncbi.nlm.nih.gov/?term=Yan+J&cauthor_id=35218676), et al.  Jinhui Liu et al.*  Yin Weijiao et al.  Jinlong Qin, et al. | Pristimerin  Sulfasalazine  Metformin  Metformin  Brusatol  Guizhi Fuling Capsule  Juglone  Juglone  Plumbagin  Simvastatin  Lovastatin, simvastatin  Sorafenib  Sorafenib  Sorafenib  Benzylserine,  GPNA  DETA/NO  Sitagliptin  -  -  -  -  -  -  -  -  -  -  Verteporfin  -  -  -  -  -  -  -  -  -  -  -  -  -  - | FANCD2  xCT  Nrf2  Nrf2  Nrf2  Nrf2  p21  HMOX1  p21  HMG-CoA  HMG-CoA  FLIP,  Mcl-1  Mcl-1  Ras/Raf/Mek/MAP  ASCT2  CDKN1A  DDP4  PTPN18  SLC7A11  p21  DDP4  p53  YAP  YAP  YAP  YAP  YAP  YAP  YAP  FSP1  HSPA5  Nrf2  Iron  Iron  Iron  CDKN1A  TUBB4A, TMPRSS2,  STX18, LINC01224,  SLC25A35,  CD7, COL23A1, ZG16B, KCNK6, NWD1, C11orf63, GZMM,  NMU  SLC7A11  MGST1  HMOX1, KEAP1, HSBP1, SAT1, CISD1, GPX4  MDM2, GPX4, PRKAA2, PRNP, SLC11A2,  ATP5MC3, PHKG2, ACO1  SLC7A11, SAT1, CDKN1A, ATP5MC3 | MTDH  -  -  IDH1-ɑ-KG-TET1  PPKCD  p62-Keap1  -  -  p53  MAPK,  AKT/mTOR  -  -  ERK/Elk-1  -  -  -  -  -  (P)RR  SOX2  -  UBE2C  -  ASPP2  p190A  -  -  -  Mst1/2-Lats1  TGF-β1  -  -  -  -  -  -  -  -  Hypermethylation  -  -  - | Hec50, KLE  USPC-1 ,  SPAC  Ishikawa,  RL95–2  Ishikawa, HEC-1-A, SPEC2, HEC-1-B,  AN3CA, ECC-1  RL95-2 ,  AN3CA, MCF7  -  Ishikawa  Ishikawa  Ishikawa  ECC-1, Ishikawa  Ishikawa  Ishikawa, KLE, RL-95/2, HEC-1A  HEC1A, HEC1B, RL95-2  -  Ishikawa, RL95-2, HEC1A, KLE  AN3CA, KLE, HEC-1B, Ishikawa  AN3CA,  HEC-1-B,KLE,  Ishikawa,  HEC-1-A  KLE  Ishikawa, AN3CA, HEC-1-A  HEC59, EN, JHUEM7  HEC-1-A ,Ishikawa  Ishikawa, RL95-2, KLE, HEC-1B  Ishikawa, RL95-2, HEC1A, AN3CA, KLE  RCB1152  RL95-2, Ishikawa, HEC-1A, HEC-1B, KLE  HEC-1-A, Ishikawa  HEC-1-A, HEC-1-B Ishikawa  KLE, EFE184  EFE184, KLE  HEC-1A,  KLE  -  -  -  -  -  -  -  -  -  -  -  - | Yes  Yes  -  -  Yes  Yes  -  -  -  -  -  -  -  -  -  -  Yes  -  Yes  -  Yes  Yes  -  -  Yes  -  -  -  Yes  -  -  -  -  -  -  -  -  -  -  -  -  - | -  -  -  -  -  -  -  -  -  -  -  -  -  Phase II Study  -  -  -  -  -  -  -  -  -  -  -  -  -  -  -  -  Retrospective study  Retrospective study  Case-control study  Prospective cohort study  Prospective cohort study  Bioinformatic analysis  Bioinformatic analysis  Bioinformatic analysis  Bioinformatic analysis  Bioinformatic analysis  Bioinformatic analysis  Bioinformatic analysis | Pristimerin reduced expression of MTDH, FANCD2 and FANCI to sensitize endometrial cancer to cisplatin  Sulfasalazine sensitized endometrial cancer to cisplatin via reducing GSH level  Metformin inhibited activation of Nrf2/AKR1C1 signaling pathway to sensitize endometrial cancer to progestin  Metformin sensitized endometrial cancer to chemotherapy by inhibit IDH1-ɑ-KG-TET1- Nrf2 loop  Phosphorylation of Nrf2 with PPKCD promoted expression of SQSTM1,which was beneficial for TAM-induced endometrial hyperplasia. Brusatol inhibited this process  GFC attenuated estrogen-induced endometrial hyperplasia through triggering ferroptosis via inhibiting p62-Keap1-NRF2 pathway  Juglone inhibited cell proliferation and promoted cell death by increasing the level of p21 and decreasing the levels of CDK2, cdc25A, CHK1  Juglone promoted expression of HMOX1, Fe2+ accumulation, lipid peroxidation and GSH depletion to induce cell death and inhibit cancer migration  Plumbagin inhibited cell proliferation and promoted cell death through up regulating the expression of p53 and p21  Simvastatin inhibited metastasis and cell proliferation through modulation of the MAPK and AKT/mTOR pathways  Lovastatin and simvastat induced cell death by targeting HMG-CoA  Sorafenib promoted proteasomal degradation of FILP and Mcl-1 to induce cell death  Sorafenib suppressed Mcl-1 at the gene transactivation level by inactivating the ERK/Elk-1 pathway to induce cell death  40 patients with carcinoma and 16 patients with carcinosarcoma was included in this study. All patients received a starting dose of 400 mg sorafenib orally twice daily. OS of patients with carcinoma was 11.4 months, PFS was 3.2 months; OS of patients with carcinosarcoma was 5.0 months, PFS was 1.8 months  ASCT2 promoted endometrial cancer cell growth by regulating glutamine uptake  DETA/NO inhibited proliferation and invasion of endometrial cancer through up-regulating CDKN1A expression  DDP4 stimulated cell proliferation, invasion and tumorigenesis. These effects were abrogated by sitagliptin  Silencing of PTPN18 might induce ferroptosis by targeting the p-p38/GPX4/xCT axis   1. RR promoted cell proliferation by inhibiting SLC7A11 expression   SOX2 specifically bound to p21 promoter DNA to inhibit p21 expression, which was correlated with histological grade and poor prognosis  DPP4 promoted cell proliferation via activation of IL-6/STAT3 signaling pathway  UBE2C enhanced p53 ubiquitination, which was associated with advanced histologic grade, FIGO stage, recurrence, invasion and shorter overall survival  YAP promoted the proliferation and drug resistance of endometrial cancer cells via regulation of IL-6 and IL-11  ASPP suppressed cell migration and invasion by reducing expression of phosphorylated YAP  p190A promoted cell proliferation and migration via activation of Hippo-YAP pathway  YAP promoted the proliferation, invasion, and migration of endometrial cancer  Increased YAP expression was associated with higher grade, stage, lympho-vascular space invasion, postoperative recurrence/metastasis  YAP/TAZ promoted the phosphorylation of IRS1 to induce insulin resistance  YAP and TAZ activated PI3K/AKT pathway signaling in endometrial cancer  TGF-beta1 stimulated cell migration and invasion by increasing FSP1 expression  The elevated expression of HSPA5 in high-risk EC tissue was found than low-risk tissue  Expression of Nrf2 was higher in endometrial serous carcinoma than other types of endometrial cancer  1,204 endometrial cancer cases and 1,212 controls were enrolled. Animal-derived iron intake was positively associated with EC risk (OR=1.9; 95%CI=1.4-2.7), predominantly after menopause (OR = 2.2; 95%CI = 1.4-3.4) and in women with BMI >or= 25 kg/m(2)(OR = 3.2; 95% CI = 1.4-7.5)  There was no association between dietary iron-related variables and risk of endometrial cancer  There was a modest positive association between heme iron, total iron, and liver intakes and endometrial cancer risk  CDKN1A was significantly associated with better prognosis of endometrial cancer  Ferroptosis associated genes were associated with prognosis of endometrial cancer. The overall survival of patients with low ferroptosis score was superior to those with high ferroptosis score  SLC7A11 was associated with TMB, MSI and drug reaction of endometrial cancer.  MGST1 was associated with poor prognosis via immune inhibition(lower levels of NK cell and CD8+ T cell infiltration)  Six ferroptosis-related genes signature was established, which was associated with prognosis and drug resistance of endometrial cancer  Eight ferroptosis-related genes signature was established, which was associated with prognosis through immune response regulation  Higher SLC7A11, SAT1 and CDKN1A expression and lower ATP5MC3 expression were linked to the low stage, grade of pTNM, and longer survival time. Many pathway was involved in this process, such as metabolism-related pathways, p53 signaling pathway |

***** Key article
